# Incorporating professional recommendations into a graduate-level statistical consulting laboratory: A case study

**DOI:** 10.1017/cts.2020.527

**Published:** 2020-08-25

**Authors:** Adam P. Sima, Viviana A. Rodriguez, Keighly E. Bradbrook, Brian S. DiPace, Victoria Okhomina

**Affiliations:** Department of Biostatistics, School of Medicine, Virginia Commonwealth University, Richmond, VA, USA

**Keywords:** Biostatistical consulting, CTSA guidelines, consulting laboratory, collaboration, graduate student, BERD, metrics

## Abstract

**Introduction:**

There has been a recent trend in medical research towards a more collaborative relationship between statisticians and clinical investigators. This has led to an increased focus on the most efficient and effective ways to structure, conduct, and measure the impact of organizations that provide statistical services to clinical investigators. Several recent guidelines and recommendations on the conduct of statistical consulting services(SCSs) have been made in response to this need, focusing on larger SCSs consisting primarily of faculty and staff statisticians. However, the application of these recommendations to consulting services primarily staffed by graduate students, which have the dual role of providing a professional service and training, remains unclear.

**Methods:**

Guidelines and recommendations, primarily from the Clinical and Translational Science (CTSA) consortium, were applied to a SCS staffed primarily by graduate students in an academic health center. A description of the organizational structure and outcomes after 3 years of operation is presented.

**Results:**

The guidelines recommended by the CTSA consortium and other groups were successfully incorporated into the graduate consulting laboratory. At almost one new project request per week, the consulting laboratory demonstrated a large bandwidth and had an excellent feedback from investigators.

**Conclusions:**

Guidelines developed for larger statistical consulting organizations are able to be applied in student-led consultation organizations. Outcomes and recommendations from 3.5 years of operation are used to describe the successes and challenges we have encountered.

## Introduction

Access to biostatistical expertise has been identified as an important step toward producing clinically impactful research [1]. Institutes of higher learning (IHL) and academic health centers (AHCs) typically provide resources for clinical researchers to obtain assistance in study design or data analysis. The size, structure, and availability of these statistical consulting services (SCSs) vary based on the institutional demands for this resource, but also with the financial and personnel resources of the IHL or AHC [2]. As institutional and grant funds intended for research support have become limited, the structures of the SCSs have become diverse, with many funded through a mixture of external, internal, and fee-based funding sources [3,4].

Regardless of this funding structure, SCSs are under increased pressure to demonstrate their scientific impact. Recently, recommendations describing the operational structure, metrics, and the evaluation of SCS have been provided [4–8]. These guidelines are intended for relatively large SCSs, including academic departments, Biostatistical, Epidemiology, and Research Design (BERD) cores of Clinical and Translational Science Awards (CTSA), and SCSs within National Cancer Institute-designated Cancer Centers (NCICC), all of which focus primarily on furthering the research profile of the IHL or AHC. However, another class of smaller SCSs, graduate consulting laboratories (GCLs), are ubiquitous in IHLs and AHCs. These organizations differ from the larger SCSs in that they focus not only on producing a statistical service but also on the training of future statisticians. GCLs vary in how graduate students participate in the research process. Students can serve as the primary consultant, an assistant to the faculty member, an observer, or as a member of a team of consultants to provide a similar service as the SCSs. GCLs are popular due to their low-operating costs coupled with the simultaneous benefit of providing students with valuable, first-hand collaborative experience, and are found in almost all statistics, biostatistics, and mathematics graduate programs and, in some instances, include undergraduates.

While the organizational structure of several SCSs has been described [9–14], there has been little to no discussion of how recent recommendations on the operational structure, metrics, and the evaluation of SCSs apply to GCLs. As such, the purpose of this article is to describe a GCL within an AHC according to the specific recommendations from the CTSA consortium and other large SCSs. We detail the organizational structure of the Virginia Commonwealth University’s (VCU) Biostatistical Consulting Laboratory (BCL), report on the projects we have been involved, and describe the feedback from our clients during the first three and a half years after reorganization. This article focuses on BCL’s impact on the research environment of our institution and leaves any discussion of educational outcomes of our consultants to a future discussion.

## Operational Structure

### BCL Overview

The VCU’s BCL provides biostatistical expertise to all researchers affiliated with VCU, including faculty, staff, fellows, residents, post-doctoral fellows, and students. Services provided by BCL are almost exclusively performed by graduate students in the Department of Biostatistics. Approximately, 4–5 students (BCL Assistants) per semester enroll in a 1-credit, weekly “Biostatistical Consulting” course dedicated toward participation in this laboratory. Other students are also involved in projects in an *ad hoc* manner according to the students’ availability and the demand for assistance. Additionally, 2–3 teaching assistants (BCL Associates) are assigned to this section to meet with investigators, assist in oversight of projects, and contribute to the administrative process of the GCL. A more detailed description of the roles and responsibilities of the BCL Assistants and Associates will be specified in subsequent subsections.

BCL is available to all investigators affiliated with VCU; however, BCL does not engage in projects related to assistance with internal or external grant applications, requests by undergraduate or graduate students related to dissertations, theses, capstone projects, or other requirements for academic advancement. Additionally, requests that BCL could not handle in a timely fashion, due to some combination of consultant availability or irresponsibly scheduled deadlines, roughly defined as any deadline within 4 weeks of the request, were not handled by the BCL staff. In each of these situations, requests are forwarded to faculty biostatisticians funded through the CTSA BERD core, the Massey Cancer Center, or other faculty members based on the area of clinical focus, biostatistical expertise, and faculty availability. Prior to forwarding the project to a faculty biostatistician, investigators requesting assistance with irresponsibly scheduled deadlines are informed that their expected timetable may not be met.

Investigators typically request BCL assistance through an online REDCap portal [15,16], which is hosted on our department’s website and advertised through a number of different institutional mailing lists. Additionally, individual faculty members in the Department of Biostatistics can request student assistance with their unfunded collaborative projects. Most students participate in the collaborative research experience as the primary biostatistician, which involves leading meetings, performing analyses, and summarizing the results in a written document. Further details on this process can be found in the following sections.

Establishing BCL was motivated by our faculty’s recognition of a lack of formal and consistent preparation for the statistical consulting practice. Additionally, with only one dedicated staff biostatistician, the hope was that a functional BCL could reduce some of the effort dedicated by faculty toward smaller, unfunded projects. Initiating our laboratory took over six months of preparation prior to accepting projects. During this time, support was provided by our department’s leadership to incorporate BCL into the academic curriculum as well as with technical aspects related to the tracking system and network storage. Protected time was allotted to develop our internal and external policies as well as implement our REDCap tracking system and network storage infrastructure. Additionally, the BCL Supervisor dedicated time to train the BCL Associates to lead projects with minimal supervision as well as guide all BCL consultants in their collaborative projects. Additional effort was spent developing the training materials to ensure the BCL Assistants were prepared to meet with investigators. An information technology professional assisted in building the REDCap tracking system as well as the network infrastructure; however, this effort has been less than 1 h per week over the course of BCL’s existence.

### Personnel

**Fig. 1.**
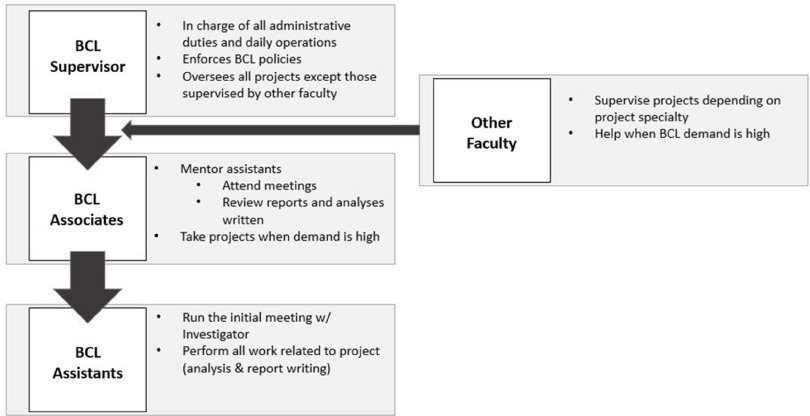
BCL staff responsibilities.

BCL has a simple organizational structure that was designed to provide supportive oversight of the student collaborators. We use a team science approach to encourage a collegial, goal-driven atmosphere that supports the dual goals of scientific achievement and graduate education [4,8]. The BCL Supervisor and Associates provide much of the project oversight and perform administrative duties, while BCL Assistants, often early in their education, serve as the primary statistical consultants (Fig. 1).

The BCL Supervisor oversees most projects, moderates the class associated with BCL, and monitors the completion of all administrative duties. Following research meetings between the consultant and investigator, the BCL Supervisor and each consultant work together to ensure a proper understanding of the background, motivation, and data, craft a statistical analysis plan (SAP), and compile a final report intended for the investigator. Administratively, the BCL Supervisor assigns projects to students, approves SAPs and final reports, monitors project timelines, and handles enforcement of BCL policies, including authorship and adherence to deadlines. This individual meets with the BCL Associates to discuss ongoing projects at least once a week, evaluates progress, plans new activities, and manages administrative duties. Other faculty members may oversee particular projects in which they have content area or biostatistical expertise but have limited administrative responsibilities.

While the BCL Supervisor can meet with investigators in a supervisory role, much of this responsibility is discharged to the BCL Associates. These individuals support the BCL Assistants in the initial research meeting with the study team and provide them with feedback and support throughout the project. Additionally, the BCL Associates review all written documents and analyses performed by the BCL Assistants prior to review by the BCL Supervisor. When there are a large number of project requests, the BCL Associates can serve as the primary consultant on projects, often with minimal oversight from the BCL Supervisor. These situations are also used by the BCL Assistants as shadowing opportunities. Administratively, these individuals are considered teaching assistants for the “Biostatistical Consulting” course.

The BCL Assistants serve as primary consultants for the majority of research projects. As such, these students are expected to act as the principal point of contact, lead the initial research meeting, and perform all statistical work. At the study conclusion, the BCL Assistants provide the study team with a written report communicating the final results or recommendations, which is approved by both the BCL Supervisor and Associate. They may also attend meetings with a BCL Associate to gain additional exposure to consultations. In most cases, the BCL Assistants are enrolled in the “Biostatistical Consulting” course, but others may participate on a volunteer and as-needed basis.

Having stable and sufficient resources for the operation of any SCS is critical for its success [17]. The BCL Supervisor receives about 0.25 full-time equivalents (FTE) dedicated to overseeing the laboratory, which comes from a mix of funds provided by the home department for teaching the “Biostatistical Consulting” course and funds from a CTSA award [4,6]. Approximately, 40 h per week of Teaching Assistant support is provided for the BCL Associates originating from the School of Medicine. Finally, the BCL Assistants are involved through the consulting course and on a volunteer basis, requiring no funding sources outside of those provided for their student status.

All BCL consultants are MS or Ph.D. graduate students in the Department of Biostatistics and have completed their first year of graduate training, which covers regression-based analysis, study design, and introductory data management topics typically associated with a first-year curriculum in biostatistics. Associates are selected through a competitive application and interview process that assesses their ability to interact with investigators and potential to contribute to the administrative and education goals of our laboratory. All consultants received some training on how to interact with investigators. Originally, this information was compressed into three 1-hour lectures embedded within “Biostatistical Consulting” course. The topics of these lectures included selected pieces of information contained in the first, second, and fifth cell of the topics described in Table 1, specifically, a description of productive collaborative relationships, strategies to achieve the productive collaborative relationships, and a discussion on the policies and procedures of the laboratory. Following feedback from the students involved in the laboratory, we extended this training to a 2-week “bootcamp” offered during the summer semester with expanded lectures on topics such as ethics and written communication. Topics included in this “bootcamp” are covered in Table 1 and are similar to those provided in the study by Taplin [18].

**Table 1.** Sample training schedule for the BCL Bootcamp

	Past “Bootcamp”				
Week 1	Description of productive collaborative relationships [19–21]	Strategies to achieve productive collaborative relationships [22–25]	Written communication skills [26,27]	Ethics [28–30]	BCL policies and procedures
Week 2	Mock consulting sessions	Mock consulting sessions	Consulting panel discussion	Review and feedback on mock consulting session	Course wrap-up
	Proposed “Bootcamp”				
Week 1	Description of productive collaborative relationships [19–21]	Strategies to achieve productive collaborative relationships [22–25]	Written communication skills [26,27]	Ethics [28–30]	BCL policies and procedures
Week 2	Video Consulting Assessment	Troubleshooting [31]	Consulting panel discussion	Efficiency and effort in consulting practice [32]	Course wrap-up

BCL, Biostatistical Consulting Laboratory

The “bootcamp” initially included video sessions of mock training sessions. While students who were involved in these sessions provided positive feedback, providing the mock consulting sessions required a large time commitment. Additionally, we observed a fair amount of angst among the students about instances when the ideal consulting relationship falls apart. Finally, qualitative feedback from the students raised a concern about the amount of effort dedicated to BCL activities. To address these needs, we plan to replace the individual mock consulting sessions with a review and discussion of a single-videotaped consulting session as well as sessions on maximizing efficiency and troubleshooting. Our proposed topics for a new iteration of the “bootcamp” are displayed in the lower panel of Table 1.

## Metrics and Project Tracking

Monitoring of the performance and impact of the SCSs are essential to demonstrate a healthy and productive organization [6,7]. With this purpose, we implemented a metric capture system in REDCap to easily obtain information on BCL’s users as well as both the effort allocated and summary data on the outcome for each project (Table 2). This system closely resembles other research tracking systems [33]. Most items collected in this system are adaptations of measures suggested in the literature for best practices in SCS in academic environments [5,7] and can be broken down into the initial request, assignment and effort reporting, closure, satisfaction, and publication information.

**Table 2.** Project information collected in the BCL tracking system

Request intake Investigator contact information Investigator affiliation Brief project description IRB/IACUC status Type of request (study design, database initiation, and data analysis) Expected deliverables (conferences, peer-review journal articles, posters, and oral presentations) Deadlines Effort tracking Type of activity (meeting, data analysis, report writing, and statistical search) Time spent on activities (hours and minutes) Closure Type of request solved (study design, IRB submission, data analysis, etc.) Type of study design (randomized clinical trial, cohort, case-control, etc.) Statistical methodology used Software used Publications Type of deliverable (peer-reviewed journal article, poster, oral presentation, etc.) Status (in preparation, under review, and accepted) Citation Satisfaction Overall satisfaction Efficiency/timeliness Professionalism Collegiality Knowledge/skill base

IRB, institutional review board; IACUC, institutional animal care and use committee

Along with information describing the clinical topic, the initial request has information about the investigators’ affiliation to the university, allowing us to assess which units have the highest demand for our resources. Some of the items that are collected can be seen in Table 2, and the actual intake form can be viewed at go.vcu.edu/BIOSConsult. This information helps the BCL Supervisor to assign the project to a faculty member or student with the necessary expertise.

Projects accepted by BCL are assigned to a primary, and in many cases, a secondary consultant who are often the BCL Assistants and Associates, respectively. BCL staff reports the time spent in meetings with the investigator, performing reviews of clinical or statistical content related to the project, analyzing data, and generating reports. When a project reaches a reasonable conclusion, a closure form that records information about the project, including the type of statistical methods used and clinical keywords, is completed by the primary consultant assigned to the project. This information is linked to a search feature that allows BCL consultants to identify projects using similar analysis strategies, allowing for completed projects to act as templates for projects requiring a similar statistical methodology. Following the completion of closure form, satisfaction surveys based on established guidelines are automatically sent to the investigators with reminders if the survey is not completed after 2 weeks [7,34]. Additionally, BCL consultants record submission of manuscripts stemming from that work and also state whether these manuscripts were eventually accepted and published. These pieces of information are periodically reviewed for all projects to ensure that any issues that arise are dealt with quickly and that our records remain current.

The periodic reviews of our operation identified recommendations for improvements to our policies, infrastructure design, and training strategy following BCL’s initiation. One major shift was changing from having a project request/tracking system form unique to BCL to one that will be utilized as the primary tracking system for our institution’s CTSA and NCICC. Since our GCL used the guidelines developed for these entities in the creation of its tracking system, many of the reporting requirements overlapped. However, incorporating the unique scientific and administrative reporting requirements for each of these organizations has been a particular challenge. At the time of this writing, the single-tracking system is in the infancy of testing.

## Project Progress

**Fig. 2.**
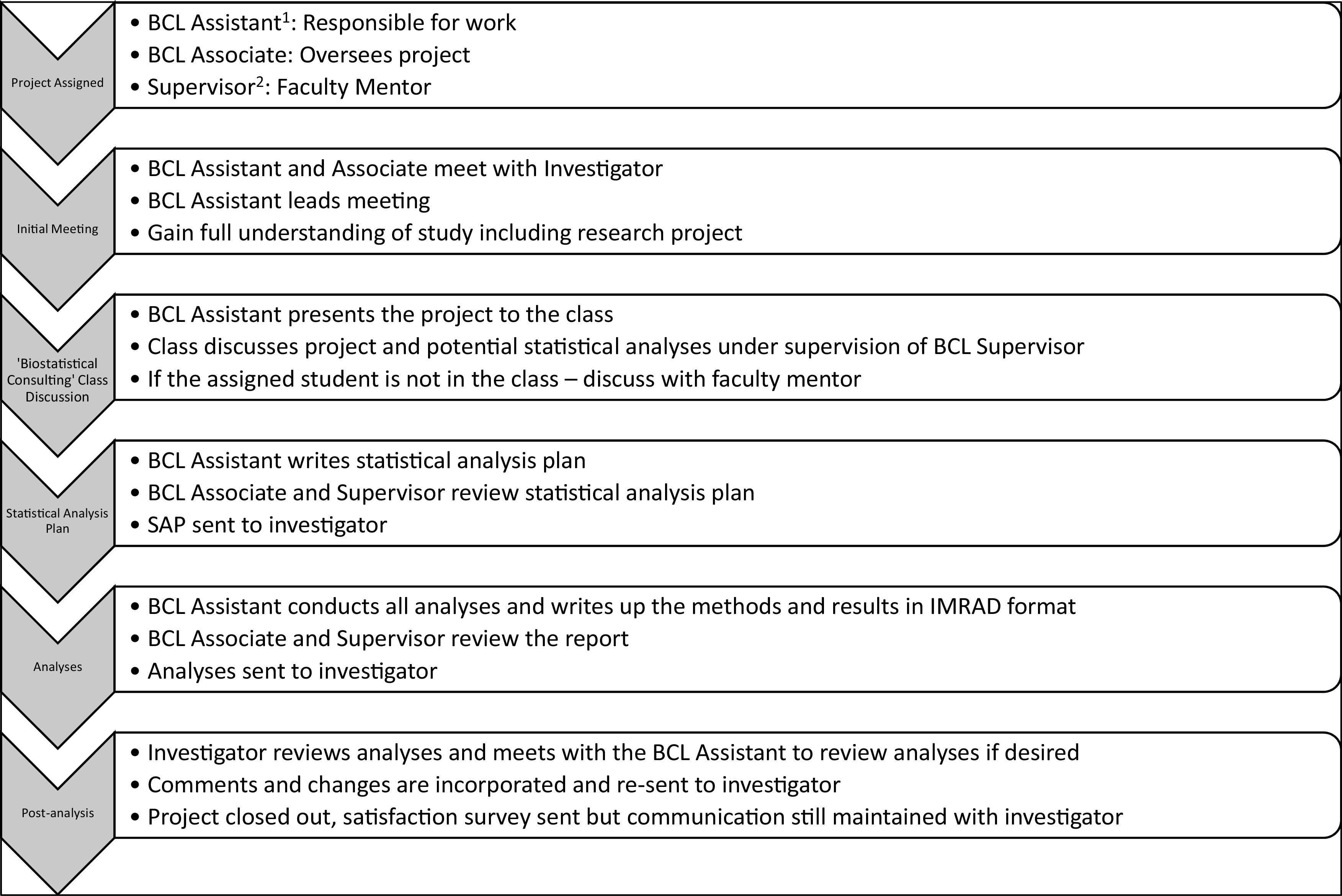
Flow chart for a typical BCL project; IMRAD: introduction, methods, results, and discussion. ^1^BCL Assistants are students enrolled in the “Biostatistical Consulting” course or volunteers wanting applied experience. BCL Associates may take these responsibilities when demand is high ^2^Supervisor is most often the BCL Supervisor, however, other faculty members may serve in this role where appropriate.

The process of a typical project is shown in Fig. 2. Once a project is assigned, the primary consultant contacts the investigators to arrange an initial meeting. All investigators are expected to meet face-to-face for an initial meeting, allowing our students to build a rapport that is supportive of a collaborative, rather than consulting, experience [17,35]. The meeting request prompts the investigator to be prepared to discuss (1) the motivation and objectives of the research, (2) the proposed methods for obtaining the data, (3) the variables and methods used to measure these variables, and (4) the deliverables that the investigator hopes to be provided at the end of the collaboration. This primary consultant is also tasked with leading the initial meeting and any follow-up meetings. Meetings with investigators take place in a variety of locations provided by the university, including investigator’s offices, conference rooms, library common areas, and even faculty lounges.

Following the initial meeting, the primary consultant provides an informal, discussion based presentation of the project to the other BCL Assistants, Associates, and the faculty advisor in the “Biostatistical Consulting” class. The primary consultant is encouraged to discuss the background, motivation, study design, and, if available, data structure to provide all individuals with enough information to understand any of the project enough to provide a statistical solution. After this discussion, a SAP is often created and sent to the investigator to outline our recommended analysis strategy. Once agreed upon, the primary consultant performs the analysis described in the SAP, with all work reviewed by the secondary consultant and BCL Supervisor. A final written report is produced by the primary consultant, reviewed and approved by the faculty mentor, and sent electronically to the investigator. Follow-up meetings to discuss the results are strongly encouraged but not mandatory.

GCLs have a relatively high turnover rate compared to most SCLs staffed with full or part-time statistical personnel. This is due to limited time students are expected to be in a program, which can range from 1 to 5 years depending on the program and intended degree. Additionally, students may not be able to continue projects indefinitely due to research assistantships, thesis/dissertation demands, or other training requirements. Therefore, it is important to create a structured system that will allow a smooth transition if the original primary consultant is unable to continue with the project. To address this, we constructed a shared network infrastructure to store all BCL consulting files securely and backed up daily. Separate folders for each project are created, each containing subfolders for data, statistical programs, reports, and other notes or communication that may be useful if a new consultant is required for a project. All BCL personnel have access to this network drive, and students are prohibited from saving any project files on their personal computers, guaranteeing the security of any protected health information. Additionally, statistical coding and report templates were created to aid the standardized organization and reporting that would be useful if students or faculty needed to provide further assistance if the original consultant was no longer available.

Instances of investigators requesting further assistance following the departure of the primary consultant have been rare at this stage of our laboratory, as only 1–2 Ph.D. classes required to participate in BCL have graduated from our department. A replacement consultant is assigned to a project 1–2 months before the original primary consultant plans on leaving. If possible, this individual is the BCL Associate or secondary consultant on the project. If a consultant has graduated, and the investigator requests additional assistance, he or she has been encouraged to continue to assist if possible. Otherwise, the BCL Supervisor takes responsibility if this individual is unable to perform this work. Issues related to manuscript submissions or addressing reviewer comments are the most common situations of an investigator requesting further effort from a consultant no longer in the department.

Since our laboratory’s initiation, we have taken steps to align our practices with modern technological advances. We have experimented with video conferencing which coincides with the increased popularity of remote statistical consulting jobs commonly found in industry settings. We have also tested our capabilities at generating reproducible reports for a variety of statistical software packages that are accessible and useful for early-stage Ph.D. and master’s students [36,37]. Recommendations on measuring our consultants’ impact on each project have been incorporated into our tracking system [5].

## Outcomes and Evaluations

Metrics from 3.5 years of BCL operation, from August 2016 to February 2020, are presented in Table 3. Roughly a quarter of BCL requests were not accepted due to the inappropriateness of the request (e.g., grant application, student thesis, or dissertation) or irresponsibly scheduled deadlines, with almost all being forwarded to faculty biostatisticians. Of the 160 requests that were accepted, the majority originated from the School of Medicine (86%), and half were requested by residents and fellows (49%). Most investigators contacting BCL were seeking assistance with data analysis (87%), while a minority made inquiry about study design (33%) and database initiation (10%). Since investigators may choose multiple services, these percentages do not sum to 100%. Requests for database initiation tended to be focused on advice on what and how to record in the study data and technical questions about starting a REDCap data collection instrument. Anecdotal evidence suggests that requests for design assistance have increased within a few subspecialties. However, the total number of design requests is small compared to those to the requests for data analysis, thus, more focus could be paid to encourage collaboration earlier in the research process.

**Table 3.** BCL metrics from August 2016 to February 2020

	N	%
**School**		
Medicine	138	86
Pharmacy	7	4
Health professions	4	3
Humanities/sciences	3	2
Nursing	3	2
Social work	2	1
VCU libraries	2	1
Center for Clinical and Translational Research	1	1
**School of Medicine department**	**N**	**%**
Internal medicine	46	33
Obstetrics and gynecology	14	10
Pediatrics	11	8
Otolaryngology	10	7
Emergency medicine	8	6
Neurosurgery	8	6
Radiology	7	5
Surgery	7	5
Anesthesiology	6	4
Orthopaedic surgery	5	4
Neurology	3	2
Other	13	9
**Position**	**N**	**%**
Resident/fellow	79	49
VCU faculty	48	30
Student	23	14
Veteran’s affairs faculty	7	4
Other	3	2
**Assistance requested** ^**a**^	**N**	**%**
Data analysis	139	87
Study design	52	33
Database initiation	16	10
Other	5	3
**Services**	**Hrs**	**%**
Analysis	1473	50
Writing	913	31
Meeting with clients	291	10
Background research	170	6
Research meeting with investigator	86	3
**Effort per project** ^*^	**N**	**%**
Less than 10 h	46	39
10 to 50 h	60	51
More than 50 h	11	9

BCL, Biostatistical Consulting Laboratory; VCU, Virginia Commonwealth University

a Investigators may choose multiple services, thus, percentages do not sum to 100%

* For the 117 closed projects

Within the 3.5-year timeframe for this study, a total of 38 graduate students worked on at least one project through BCL. Thirty of the graduate students were Ph.D. students with the remaining eight seeking an MS degree. Each BCL Assistant worked on an average of 3.1 projects, ranging from 1 to 10 projects per consultant. On an average, BCL Associates led 12.5 projects and oversaw 15 others over the study timeframe. By the end of the study period, 117 (73%) projects were completed, with 51% requiring moderate effort (10–50 h) to complete. The consultants devoted an average of 21 h of work on each completed project, with about half of this time (11 h) spent performing the statistical analysis. Writing final reports was the next most time-consuming activity, with students spending 7 h (31%) of the total project time producing the report. This included the original draft as well as responding to BCL Associate and Supervisor comments.

As of this writing, 83 (52%) of the projects had submitted results as a conference presentation or peer-reviewed journal article, and 21 of those 83 projects had more than 1 submission. These projects resulted in 30 manuscript submissions (16 under review and 14 accepted) and 52 conference presentations (15 under review and 37 accepted), all with BCL consultants included as an author. Overall, feedback from investigators has shown that 77% were very satisfied with services received, and 99% considered that they received a deliverable that addressed their request (Table 4). However, timeliness could be improved (12% of investigators were either neutral or dissatisfied). We believe this item is lower, in part, due to unrealistic expectations of how long a data analysis should take. Issues with data management, particularly pre-analysis data preparation, may also contribute to a later completion date. Even with concerns about timeliness, over 97% of investigators reported that they would recommend the service to others.

**Table 4.** Investigators’ satisfaction with BCL services from August 2016 to February 2020

Feedback	Very satisfied %	Satisfied %	Neutral %	Dissatisfied %	Very dissatisfied %
Overall satisfaction with the service received	82	16	2	0	0
	**Strongly agree** **%**	**Agree %**	**Neutral %**	**Disagree %**	**Strongly disagree %**
Received statistical support that addressed my request	71	27	2	0	0
Assisted in a timely manner	64	22	11	2	0
Treated with courtesy and respect	82	18	0	0	0
Received a deliverable that addressed the request	73	24	2	0	0
The BCL consultant(s) was(were) actively engaged in the research meeting	78	22	0	0	0
Recommend the BCL to colleagues	80	18	0	2	0

BCL, Biostatistical Consulting Laboratory; VCU, Virginia Commonwealth University

A total of 45 of the 85 closed projects had a completed satisfaction survey

## Discussion and Conclusion

Involving graduate students in a professional SCS is an important goal for statistics and biostatistics departments, as it contributes to the research profile of the AHC or IHL while simultaneously preparing students for professional employment. It is crucial to not only teach the interpersonal skills used in consulting meetings but to offer training and experience using the best practices of project management. Over a 3.5-year period, our BCL has demonstrated that the indicators of best practice intended for large SCSs can be successfully applied to smaller GCLs with few resources outside of those already provided by most IHLs or AHCs. Our success as a SCS has not only been demonstrated by excellent feedback from the clinical investigators but also by an impressive track record of productive relationships as measured by authorship on a number of conference abstracts and peer-reviewed publications. This has been achieved using minimal personnel resources, which is important as financial resources for research infrastructure have been decreasing in recent years[38].

The design and conduct of BCL was based on existing recommendations for operating SCSs. Of the many recommendations, we found those by Rubio et al. [7] to be the most impactful. These recommendations were the basis of the information collected in the REDCap tracking system. Metadata from this system allows us to periodically assess project progress and demonstrate the productivity to justify BCL’s continued operation. Additionally, we found having an organized, shared network workspace with consistent coding and reporting templates to be the most impactful aspect of BCL for its day-to-day operation. Thus, we recommend that if a new GCL is to be created or restructured, focusing on both the metric tracking system and infrastructure contributing to a standardized workflow be the focus of this endeavor.

While we are proud of the achievements thus far, we continue to seek improvements in our operations. We have tested our capabilities at generating reproducible reports for a variety of statistical software packages that are accessible and useful for early-stage Ph.D. and master’s students [36,37]. A measurement of BCL’s impact on each project has been incorporated into our tracking system [5]. But most of the improvements we have explored, and will continue to explore, revolve around striving to complete projects in an efficient manner. This should benefit the consultants who need to focus their effort not only on their collaborative skills but also on their requirements for graduation, as well as the investigators who receive the results from their studies in a timely manner. We have investigated small changes, such as having the BCL consultants submit a proposed timeline for project completion to the BCL supervisor and having share to-do lists. Additionally, more substantial changes in our reporting that should decrease the time spent reporting commonly used analyses have been explored [39–42].

Like any service organization, we have identified several areas that may improve our service by more efficiently balancing resources and demands. Because BCL services are limited by the number of students available, high effort investigators must be overseen carefully. High effort investigators have multiple overlapping and/or sequential projects, or present projects with a scope of work beyond what BCL is able to provide in a reasonable timeframe. While these investigators provide our consultants with numerous opportunities for collaboration, they can overwhelm the limited amount of available effort of graduate consultants. Proper utilization of the tracking system is also an ongoing challenge. In our experience, having students keep a detailed accounting of their efforts are very uncommon. Due to this unfamiliarity, the metrics presented in Table 3 may not accurately reflect the efforts of the past 3.5 years due to the expected underreporting of effort by BCL Assistants and Associates.

One particular challenge that we, as well as many other statistical consulting organizations, have faced is investigators making requests with tight deadlines. These requests are particularly burdensome as they tend to monopolize the consultants’ time at the expense of other ongoing projects or responsibilities. Since our consultants are all students and, thus, have important educational responsibilities while simultaneously carrying a relatively high project load associated with BCL, we chose to offload the responsibility of these requests to faculty members. The strategy used to accommodate these requests varied based on the individual faculty member taking over the project, the clinical investigators, and the nature of the project/deadline itself. Anecdotally, solutions to handle these situations ranged from an agreement to work on the project, but not until after the deadline due to availability issues, to a decision to work on a simple part of the research question for an abstract submission and a more thorough investigation when time permitted, to a complete analysis meeting the inverstigator’s request. However, no unified strategy existed to handle these requests. Having a consistent policy or strategy amongst the various SCSs to alleviate this burden could be a more efficient manner to handle requests with tight deadlines.

While statistical and biostatistical faculties have a great deal of control over the policies and operation of the GCL, a number of other factors may limit its impact within an IHL or AHC. In the statistical framework, consulting is defined as a short-term interaction between a clinical investigator and statistician. On the other hand, collaboration is defined as a closer working relationship that requires equal input from all personnel [8,17]. Even though BCL consultants are trained to provide a service as if they were in long-term collaborations, very few of the projects involved our input in the early stages of study design. This limited our contribution to this crucial stage of study planning [43,44].

The study by Vance introduced a paradigm to assess research impact of a consulting organization [17]. In this structure, the lowest level (Level 0) described an organization whose focus is strictly on education. Impact Level 1 describes a successful consulting organization whose investigators’ satisfaction initiates a cycle of more successful collaborations. Level 2 is characterized by a realization from the faculty that students’ training is improved, coupled with increased funding for the organization as a recognition, it is a vital component to the research capacity of the IHL or AHC. Level 3 describes a consulting organization that is able to provide a societal impact outside of the IHL or AHC it is housed. The qualitative and quantitative feedback from both the investigators who have used BCL and faculty within our department suggests we have reached impact Levels 1 or 2. However, the continued productivity of our organization could be limited or adversely affected without additional external financial commitments. Thus, we advocate IHL and AHCs, either through or in conjunction with larger SCSs, provide joint financial support for GCLs with the academic department in which the GCL is housed. We expect this funding paradigm would eliminate the barrier that prevents our BCL from having greater impact within and outside of our AHC.

Finally, this article focused solely on the scientific or statistical contributions of our GCL to our AHC. This ignores the other important facet that is common to all GCLs, namely, the successful training of a future generation of statistical collaborators. We recognize that productive and successful GCLs should incorporate training metrics along with their scientific achievements. This discussion is beyond the scope of this article and could be addressed as future work.
